# Supplementary material for: PARP1 and PARG Are the Draft Horses for Polycomb-Trithorax Chromatin Regulator Machinery
Source: Biomolecules. 2025 Sep 12;15(9):1314. doi: 10.3390/biom15091314 (PMC12467497; doi:10.3390/biom15091314)
Supplement: Supplementary file 1 [file biomolecules-15-01314-s001.zip › Supplemental Data.pdf]

SUPPLEMENTARY APPENDIX FOR

PARP1 and PARG are the draft horses for Polycomb-Trithorax chromatin regulator machinery

Authors: Guillaume Bordet[1] and Alexei V. Tulin[1]*

Affiliations:
[1]University of North Dakota, Grand Forks, ND

THIS FILE INCLUDES:

Supplemental Figure S1. Histone peptide array blot.

Supplemental Figure S2. Mono-Methylated Histone Marks control PARP1 Binding Patterns *In Vivo*.

Supplemental Figure S3: Boxplot analysis of mono-methylated histone marks and PARP1 occupancy *in vivo*.

Supplemental Figure S4. Differential distribution of mono-methylated histone marks correlates with gene expression levels.

Supplemental Figure S5. PARP1 and mono-methylated histone marks are spread along the gene body of active genes.

Supplemental Figure S6. Mono-methylated histone marks are associated with TrxG.

Supplemental Figure S7. Boxplot analysis of mono-methylated histone marks and PARP1 occupancy at TrxG- and PcG-positive loci *in vivo*.

Supplemental Figure S8. PARP1 and Ash2 promote the expression of the same set of developmental genes.

Supplemental Figure S9. Boxplot for PARP1 and TrxG co-occupy active while PARG and PcG co-occupy repressed loci.

Supplemental Table S1 Legend: Histone peptide array raw data.

A

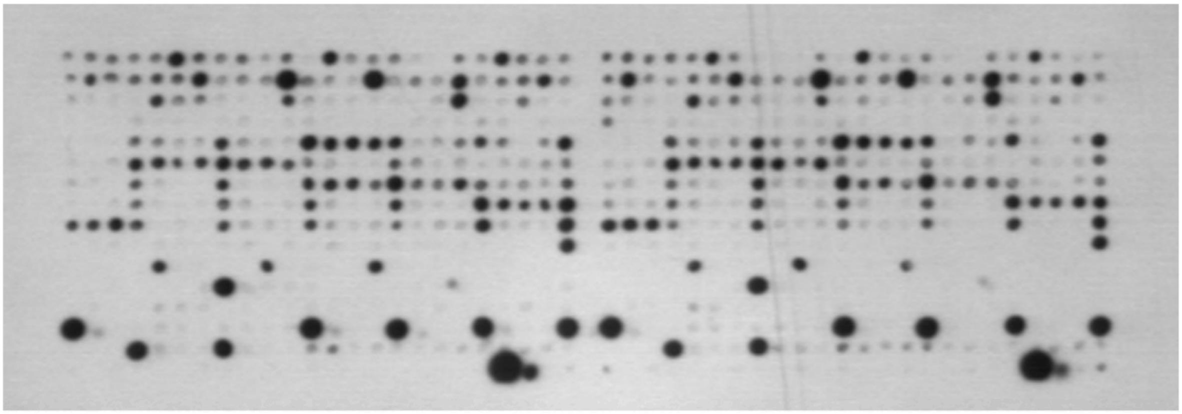

B

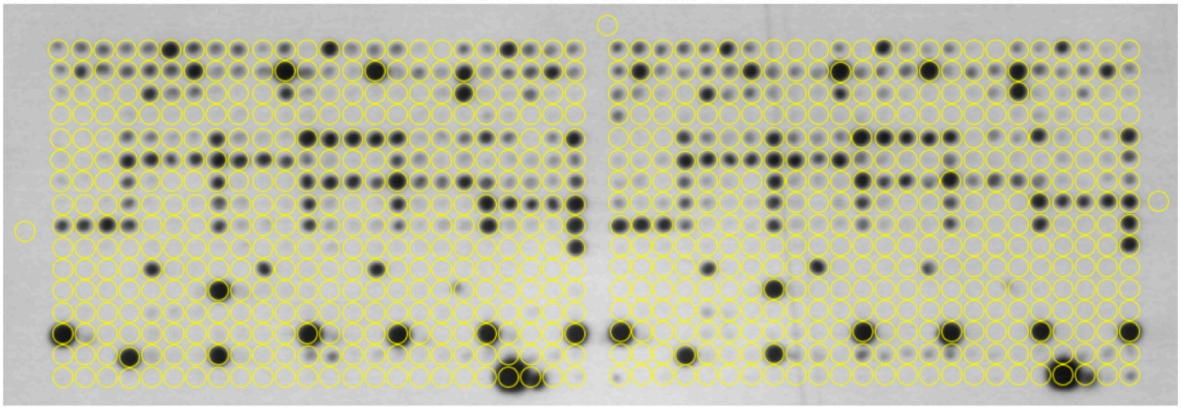

Supplemental Figure S1: Histone peptide array blot. A. Photograph of the original blots. The left and right panels represent biological replicates. **B.** Schematic illustration highlighting the regions of interest (ROIs) used for blot analysis. Signal intensity was measured within the designated ROI on the array. Background intensity was calculated as the average signal from three ROIs located outside the array (one on the left, one on the right, and one at the top). ROI were generated via Fiji software.

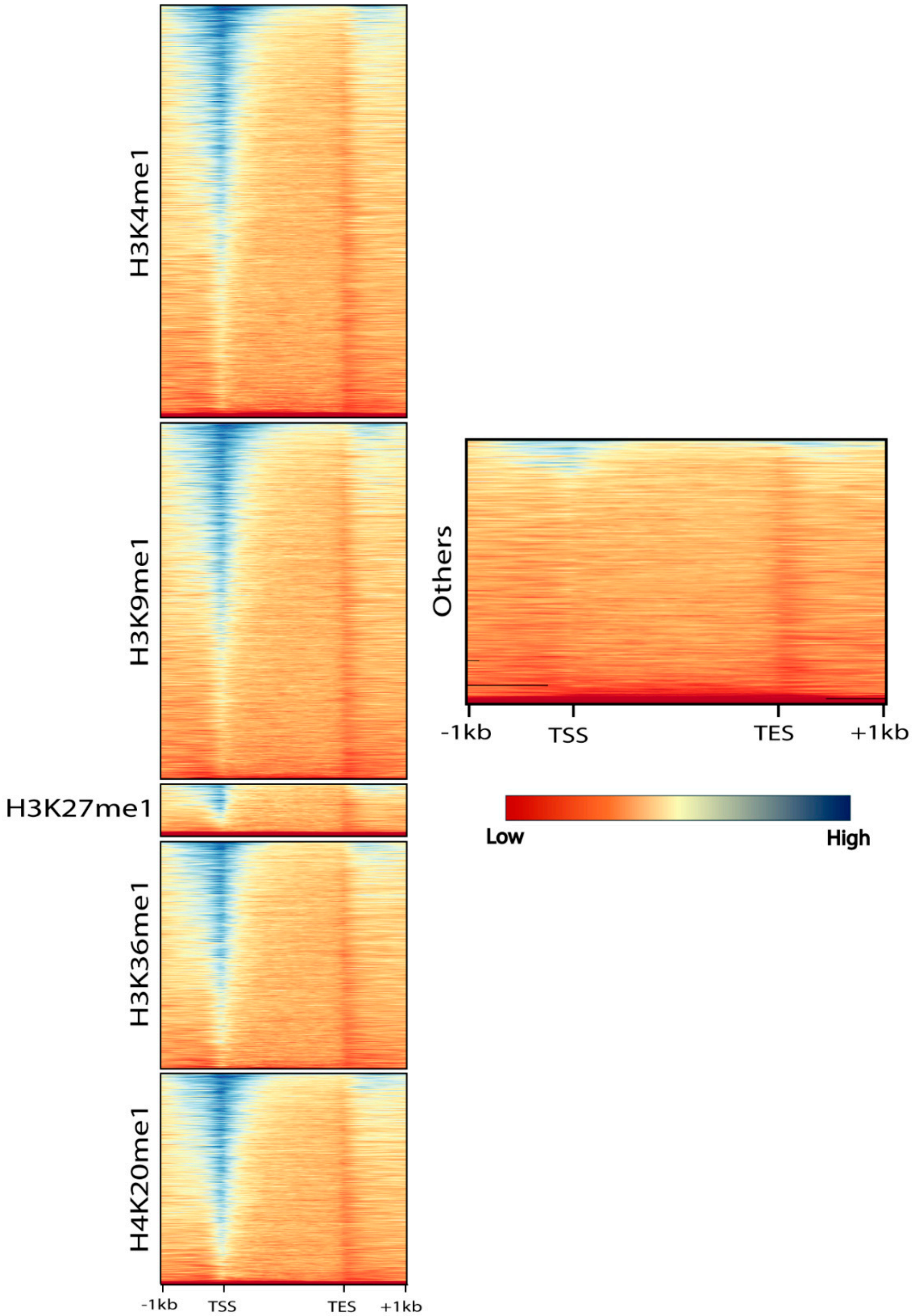

Supplemental Figure S2: Mono-Methylated Histone Marks control PARP1 Binding Patterns *In Vivo*. Heatmaps illustrating the spatial distribution of PARP1 across the gene bodies of genes positive for H3K4me1, H3K9me1, H3K27me1, H3K36me1, or H4K20me1 mono-methylated histone marks (Left panel) or across the genes bodies of the loci lacking these mono-methylated histone marks (Right panel). Color gradients range from red (lowest occupancy) to blue (highest occupancy).

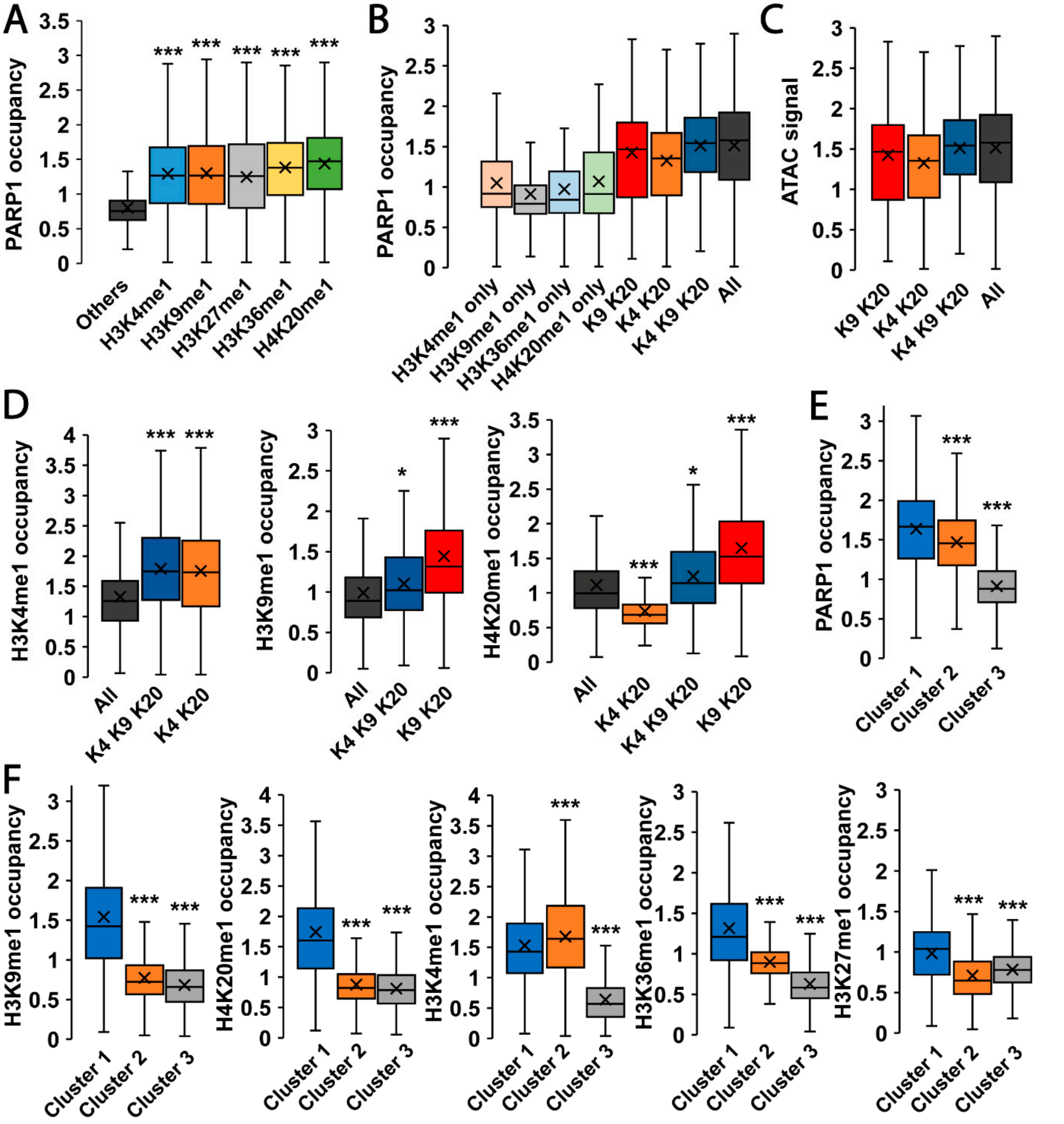

Supplemental Figure S3: Boxplot analysis of mono-methylated histone marks and PARP1 occupancy *in vivo*. A-F. The Boxplots correspond to panels in Figure 3. Boxplots display the distribution of values (25th–75th percentile), whiskers represent the range, and the y-axis indicates factor occupancy (see *Materials and Methods* for details). All statistical tests are homoscedastic two-tailed *t*-tests. (A) Related to Fig. 3A, *t*-tests are compared to the "Others" group; (B) related to Fig. 3B; (C) related to Fig. 3D; (D) related to Fig. 3G, *t*-tests are compared to the "All" group; (E) related to Fig. 3H, *t*-tests are compared to the "Cluster 1" group; (F) related to Fig. 3J–K, *t*-tests are compared to the "Cluster 1" group. ***: p-value < 0.01; *: p-value < 0.05.

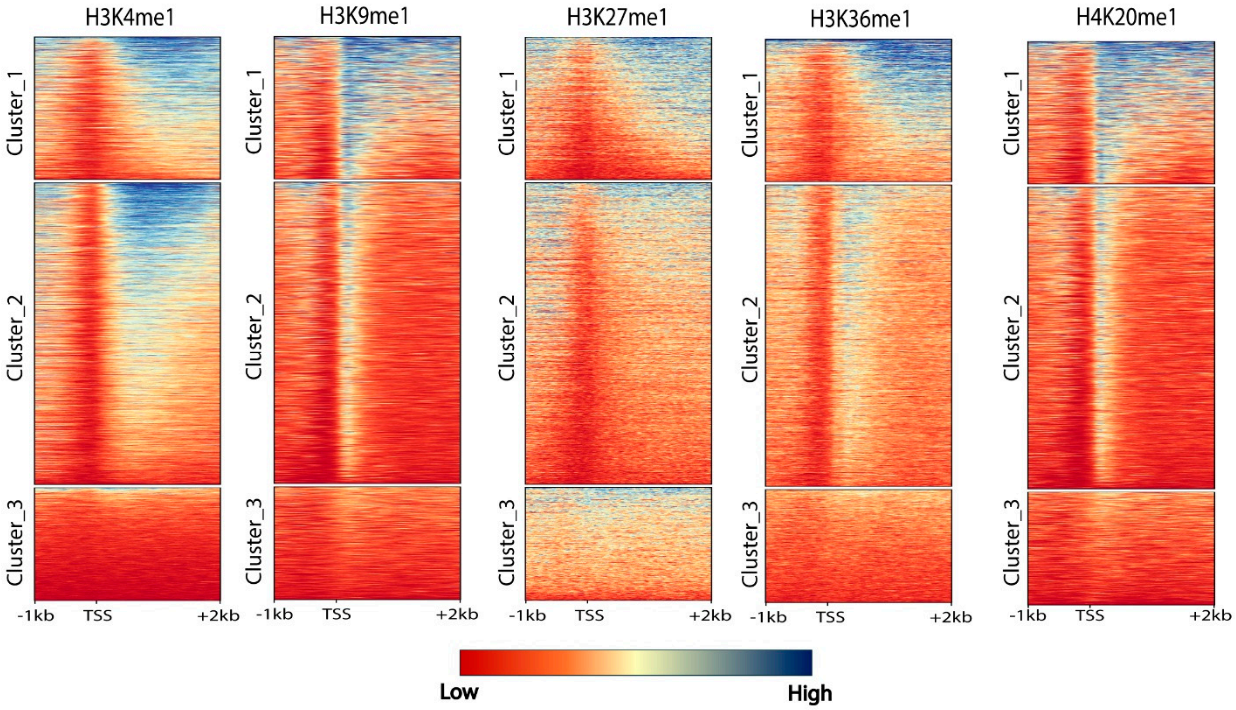

Supplemental Figure S4: Differential distribution of mono-methylated histone marks correlates with gene expression levels. Heatmaps illustrating the spatial distribution of mono-methylated histones across the gene bodies of Cluster 1 (highly expressed), Cluster 2 (moderately expressed), and Cluster 3 (lowly expressed) genes. The color gradients range from red (lowest occupancy) to blue (highest occupancy). Mono-methylated histones analyzed include H3K4me1, H3K9me1, H3K27me1, H3K36me1, and H4K20me1.

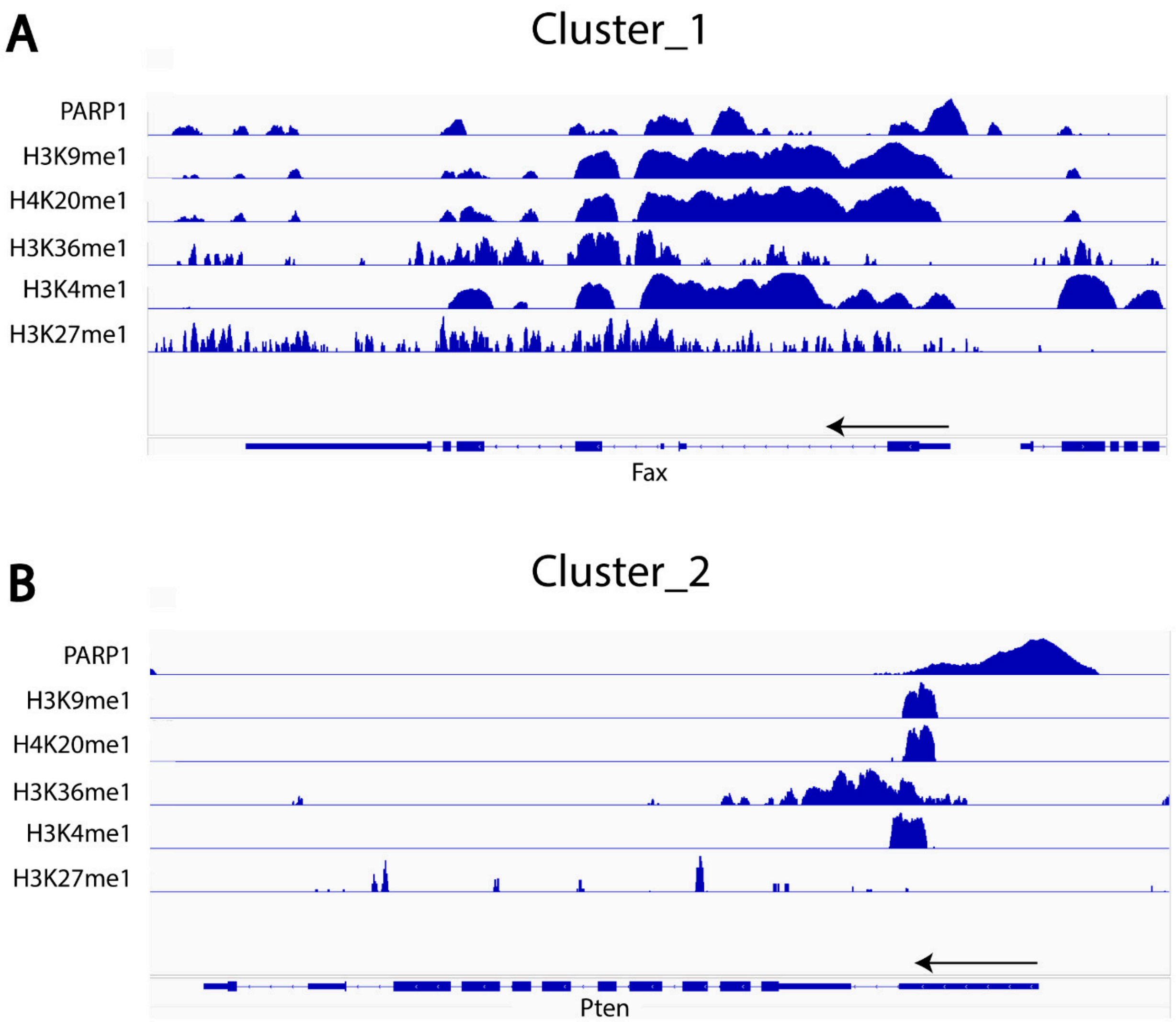

Supplemental Figure S5: PARP1 and mono-methylated histone marks are spread along the gene body of active genes. Integrated Genome Viewer (IGV) tracks illustrating the distribution of PARP1 protein and the mono-methylated histone marks H3K9me1, H4K20me1, H3K36me1, H3K4me1, and H3K27me1, along two example loci: 1) Fax (**A**), a Cluster 1 gene that is highly expressed during the wandering third instar larval stage, 2) Pten (**B**), a Cluster 2 gene that is moderately expressed during the same stage. The arrows indicate the orientation of the genes. For the highly expressed Cluster 1 gene Fax, PARP1, H3K9me1, and H4K20me1 are spread along the gene body, while H3K4me1, H3K36me1, and H3K27me1 are enriched deeper in the gene body. Conversely, for the moderately expressed Cluster 2 gene Pten, and PARP1 are present only at the promoter region, with mono-methylated histone marks sequentially enriched along the gene body: H3K9me1 and H4K20me1 are closest to the promoter, followed by H3K4me1, and then H3K36me1. H3K27me1 is depleted from the Pten locus.

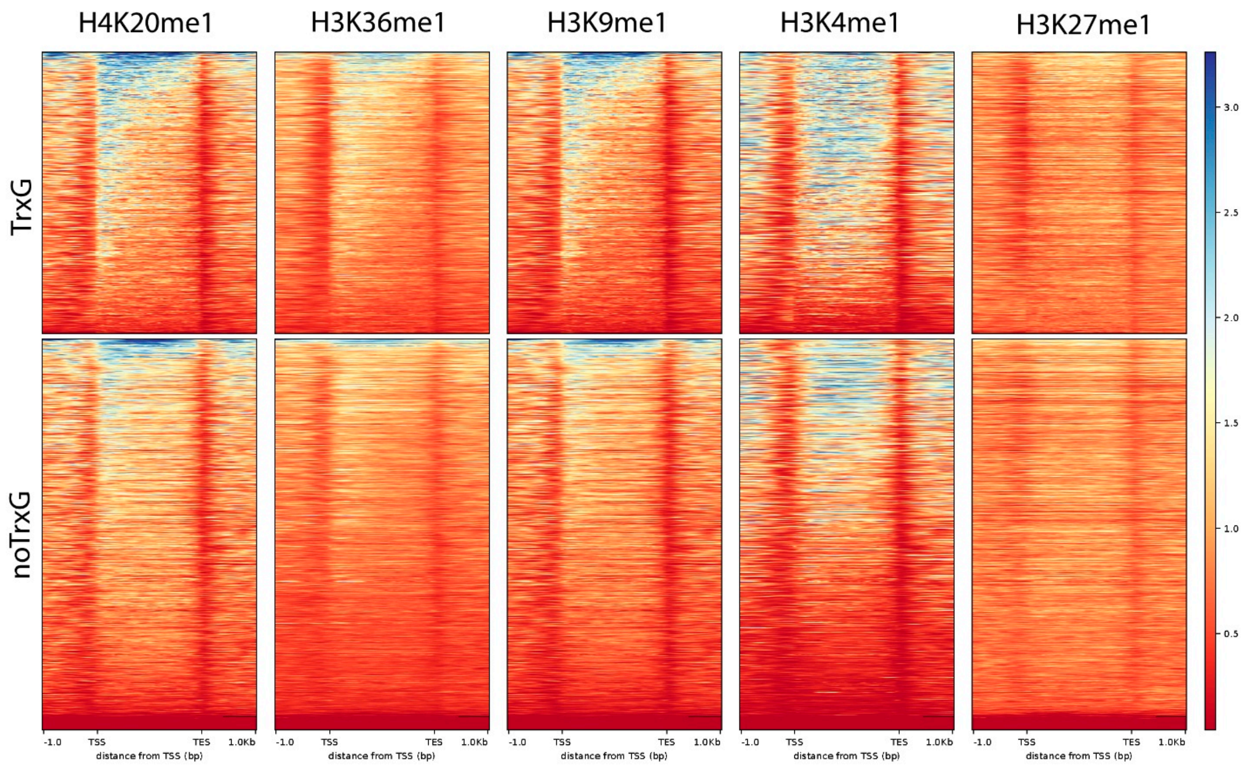

Supplemental Figure S6: Mono-methylated histone marks are associated with TrxG. Heatmaps illustrating the spatial distribution of H3K4me1, H3K9me1, H3K27me1, H3K36me1, or H4K20me1 mono-methylated histone marks across the genes bodies of the loci positive (TrxG) or negative (noTrxG) for TrxG. Color gradients range from red (lowest occupancy) to blue (highest occupancy). Top panels display the average distribution.

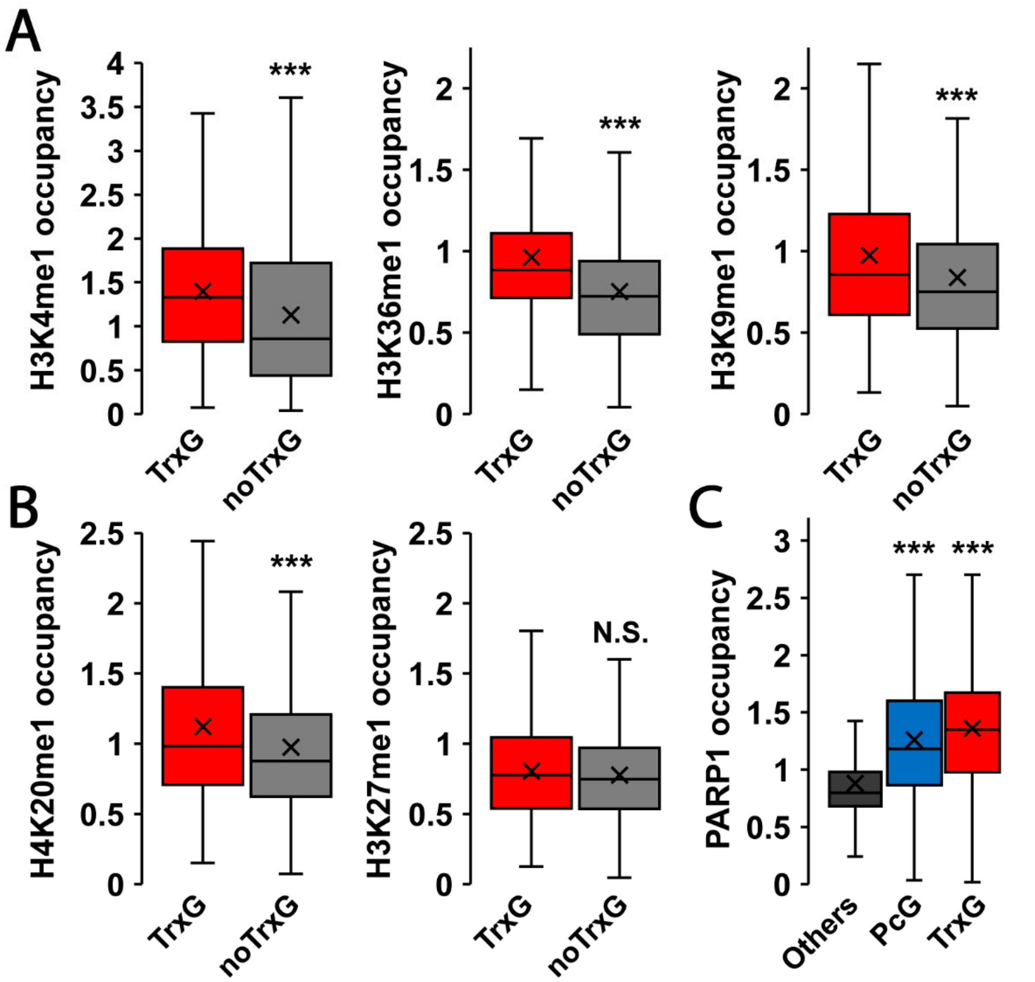

Supplemental Figure S7: Boxplot analysis of mono-methylated histone marks and PARP1 occupancy at TrxG- and PcG-positive loci *in vivo*. A-C. Boxplots correspond to panels in Figure 4. Boxplots show the distribution of values (25th–75th percentile), whiskers indicate the range, and the y-axis represents factor occupancy (see *Materials and Methods* for details). Statistical significance was assessed using homoscedastic two-tailed t-tests. (A–B) Correspond to Fig. 4A, with comparisons made to the "TrxG" group; (C) corresponds to Fig. 4D, with comparisons made to the "Others" group. ***p < 0.01; N.S., not significant.

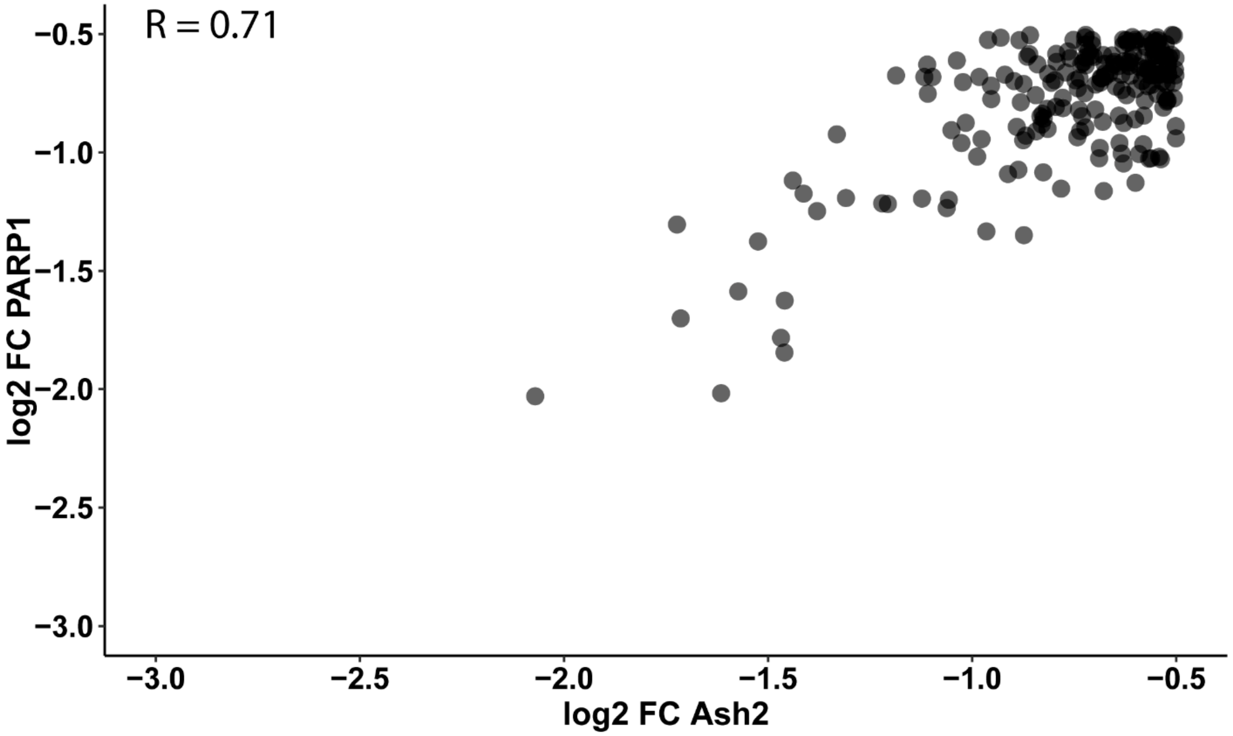

Supplemental Figure S8: PARP1 and Ash2 promote the expression of the same set of developmental genes. Scatter plot illustrating the expression profiles of the 194 developmental genes shown in Figure 4F. All these genes are downregulated in both *ash2* mutants (*ash2I1*) and *parp1* hypomorphic conditions during the wandering third instar larval stage (puff stage 7–9). The correlation coefficient (r = 0.71) indicates a strong positive relationship. Correlation was calculated by comparing log2 fold changes in gene expression between wild type and *ash2* mutants with those between wild type and *parp1* knockdown animals at the same developmental stage. Fold change was calculated from RPKM values in wild type versus knockdown conditions.

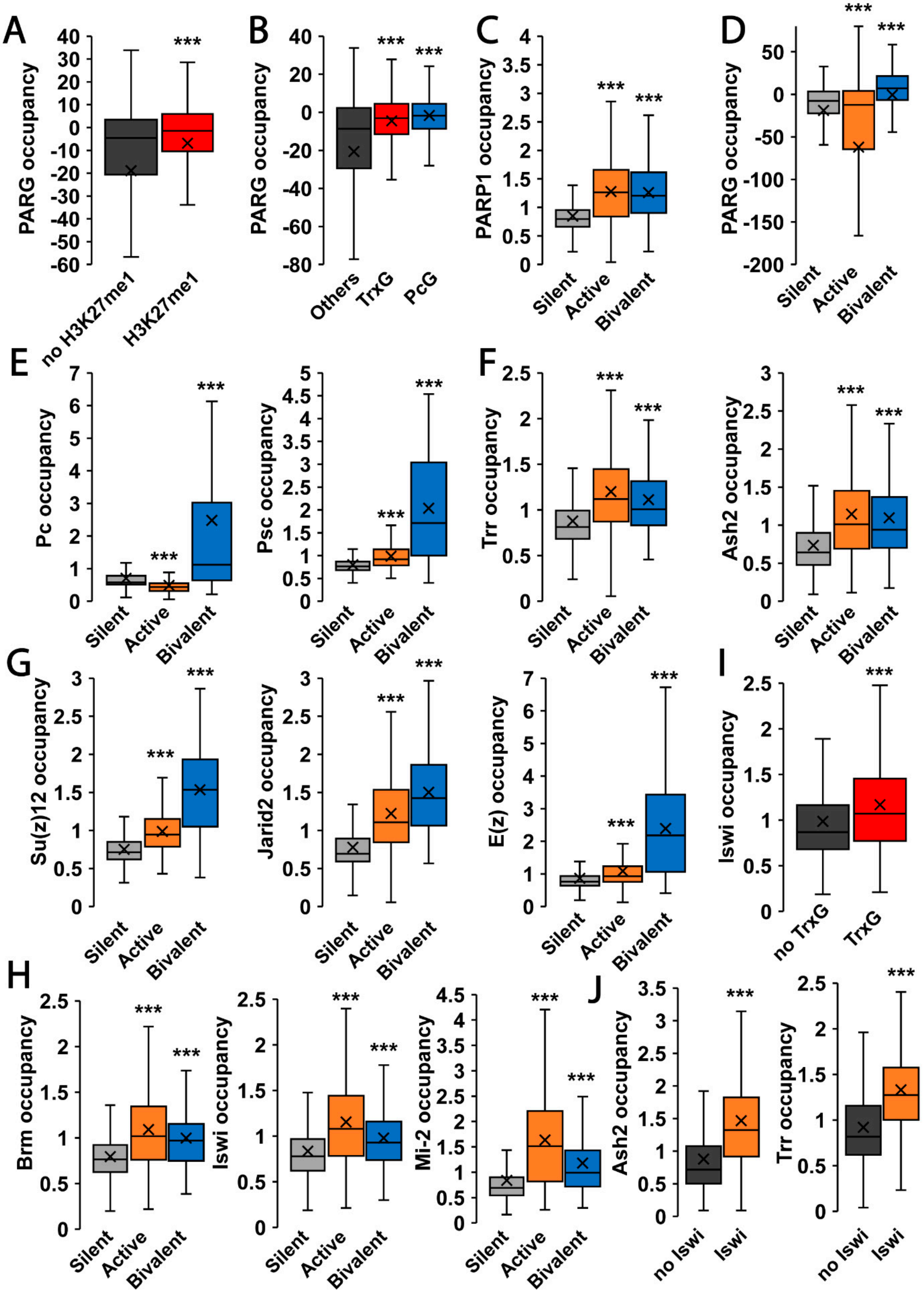
A
PARG occupancy
no H3K27me1
H3K27me1
B
PARG occupancy
Others
TrxG
PcG
C
PARP1 occupancy
Silent
Active
Bivalent
D
PARG occupancy
E
Pc occupancy
Psc occupancy
F
Trr occupancy
Ash2 occupancy
G
Su(z)12 occupancy
Jarid2 occupancy
E(z) occupancy
I
Iswi occupancy
no TrxG
TrxG
H
Brm occupancy
Iswi occupancy
Mi-2 occupancy
J
Ash2 occupancy
Trr occupancy
no Iswi
Iswi
***

Supplemental Figure S9: Boxplot for PARP1 and TrxG co-occupy active while PARG and PcG co-occupy repressed loci. A-J. Boxplots correspond to panels in Figure 5. Boxplots show the distribution of values (25th–75th percentile), whiskers indicate the range, and the y-axis represents factor occupancy (see *Materials and Methods* for details). Statistical significance was assessed using homoscedastic two-tailed t-tests. (A) Corresponds to Fig. 5A, with comparisons made to the “no H3K27me1” group; (B) corresponds to Fig. 5B, with comparisons made to the “Others” group; (C–H) correspond to Fig. 5D–I, with comparisons made to the “Silent” group; (I) corresponds to Fig. 5J, with comparisons made to the “no TrxG” group; (J) corresponds to Fig. 5K–L, with comparisons made to the “no Iswi” group. ***$p < 0.01$; N.S., not significant.

Supplemental Table S1 Legend: Histone peptide array raw data. Spreadsheet of the raw data obtained during the histone peptide array analysis. Each array consists of 16 rows labeled A to P and 24 columns labeled 1 to 24. The columns are described as follows:

- **Column 1 ("Location")**: Position of the spot on the array.
- **Column 2 ("Name")**: Information about the histone tail segment at this spot.
- **Columns 3 to 6 ("Mod1", "Mod2", "Mod3", "Mod4")**: Details of the modifications on the histone tail segment at this spot.
- **Columns 7 and 8 ("Array1", "Array2")**: Intensity of PARP1 binding at this spot, with background intensity subtracted, for Arrays 1 and 2, respectively.
- **Column 9 ("Average signal")**: Average intensity of the two arrays for this spot.
- **Column 10 ("SEM")**: Standard error of the mean (SEM) between the two arrays.
